# Editorial: Seagrasses Under Times of Change

**DOI:** 10.3389/fpls.2022.870478

**Published:** 2022-04-28

**Authors:** Gidon Winters, Mirta Teichberg, Hauke Reuter, Inés G. Viana, Demian A. Willette

**Affiliations:** ^1^Dead Sea and Arava Science Center (DSASC), Masada National Park, Mount Masada, Israel; ^2^Eilat Campus, Ben-Gurion University of the Negev, Hatmarim Blv, Eilat, Israel; ^3^Leibniz Centre for Tropical Marine Research GmbH (ZMT), Fahrenheitstraße 6, Bremen, Germany; ^4^The Ecosystems Center, Marine Biological Laboratory Starr, Woods Hole, MA, United States; ^5^Faculty for Biology and Chemistry, University of Bremen, Bremen, Germany; ^6^Instituto Español de Oceanografía (IEO-CSIC), Centro Oceanográfico de A Coruña, A Coruña, Spain; ^7^Biology Department, Loyola Marymount University, Los Angeles, CA, United States

**Keywords:** seagrasses, climate change, eutrophication, responses of seagrasses to single and combined stressors, spatial-temporal modeling

Awareness of the ecological importance of seagrasses is growing due to recent attention to their role in carbon sequestration as a potential blue carbon sink (Fourqurean et al., 2012; Bedulli et al.), as well as their role in nutrient cycling (Romero et al., 2006), sediment stabilization (James et al., 2019), pathogen filtration (Lamb et al., 2017), and the formation of essential habitats for economically important marine species (Jackson et al., 2001; Jones et al.). Despite their importance and the increasing public and scientific awareness of seagrasses, simultaneous global (e.g., ocean warming, increase in frequency and severity of extreme events, introduction and spread of invasive species) and local (e.g., physical disturbances, eutrophication, and sedimentation) anthropogenic stressors continue to be the main causes behind the ongoing global decline of seagrass meadows (Orth et al., 2006; Waycott et al., 2009).

Degradation of seagrass ecosystems entails the loss of the associated biota, primary productivity, and local fisheries, and increased sediment re-suspension and beach erosion, processes that result in severe ecological and socio-economic consequences not only for seagrass meadows but also for neighboring ecosystems and human inhabitants (Erftemeijer and Lewis, 2006; Joseph et al., 2019; Moksnes et al., 2021).

Will climate change exert diverging effects on different seagrass species? Will ocean warming eventually exceed the adaptive potential of local seagrass species resulting in a shift of their biogeographic ranges? Does eutrophication cause similar stress as exposure to thermal stress? Do seagrass populations with different “histories” respond differently to stress? Can we suggest new improvements for conservation and management of local meadows that will enhance resilience to the predicted and unpredicted scenarios of change?

In this issue dedicated to seagrasses under times of change, we have collected 17 studies authored by 104 seagrass researchers from around the world that are trying to answer many of these and other questions.

In this Research Topic, readers will find studies that compare the responses of seagrasses to single and combined stressors in their environment and their interactions through multi-stressor laboratory experiments, field studies, and spatial-temporal modeling, ranging from the cellular level (Nguyen, Yadav et al.) to ecosystem processes (e.g., Helber, Procaccini et al.; Helber, Winters et al.).

We are particularly happy to see the diversity of the seagrass studies presented here. This includes:

1. Species diversity (Figure 1): While research efforts have traditionally focused on native (usually temperate; blue bars in Figure 1) “flagship” seagrass species (e.g., *Posidonia oceanica, P. australis, Zostera marina, Z. muelleri*), this collection includes studies on a total of 13 different seagrass species, and a large proportion of the publications here are on tropical seagrass species (*Thalassia, Halophila, Cymodocea, Enhalus*; red bars in Figure 1).
2. Study type (Figure 2): Although this is a collection of only 17 publications focused on studying seagrass under changing times, this collection represents a diverse group of types of studies. The number of experimental studies (~60% of studies in this issue) goes to show that we have gone way beyond just documenting the loss of seagrasses, and much effort is going into understanding the mechanisms and improving our ability to predict changes.

**Figure 1:**
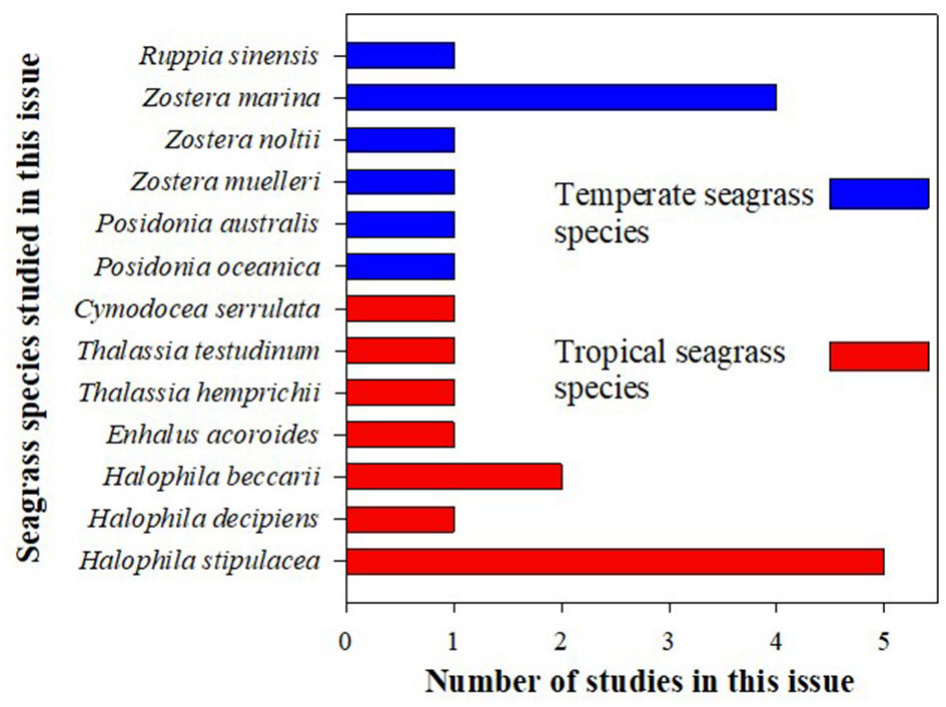
The number of species that were studied in this issue. Blue and red bars represent temperate and tropical species, respectively.

**Figure 2:**
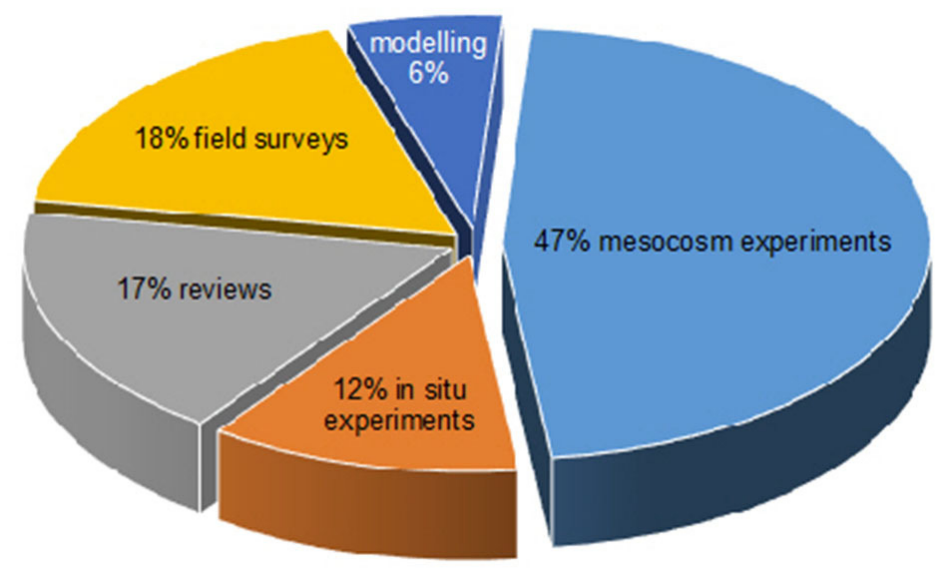
The types of studies that were published in this issue.

Mesocosm simulations (i.e., single or multi-stressor experiments under controlled laboratory conditions) account for half the studies (Artika et al.; Fang et al.; Lowell et al.; Nguyen, kim et al.; Nguyen, Yadav et al.; Premarathne et al.; Viana et al.). These mesocosms allowed authors to study seagrass responses under the combination of thermal and nutrient stress much more realistic scenarios than previous efforts that focused usually only on one stressor. In addition to the use of mesocosms, we present here papers using modeling (Beca-Carretero et al.; Rock and Daru), *in situ* nutrient manipulations (Helber, Procaccini et al.; Helber, Winters et al.), field surveys (Bertelli et al.; Duffin et al.; Gu et al.), and reviews of historical data (Green et al.; Winters et al.) that have identified gaps of knowledge that can direct future efforts (Rock and Daru; Winters et al.). While molecular studies have emerged as “standard use” in recent years, at least in the field of seagrasses, epigenetics is rather a new field (Jueterbock et al.; Nguyen, kim et al.), and the early exposure of seeds to ocean acidification (Lowell et al.) offers some practical potential in the field of restoration. Studies also include some promising new methods such as thermal priming (Nguyen, kim et al.) that offer some future potential in active restoration efforts (e.g., “super seagrasses”).

As the word cloud (Figure 3) shows, we still have many topics which at least in this issue, did not get enough attention (i.e., the small-sized words).

**Figure 3:**
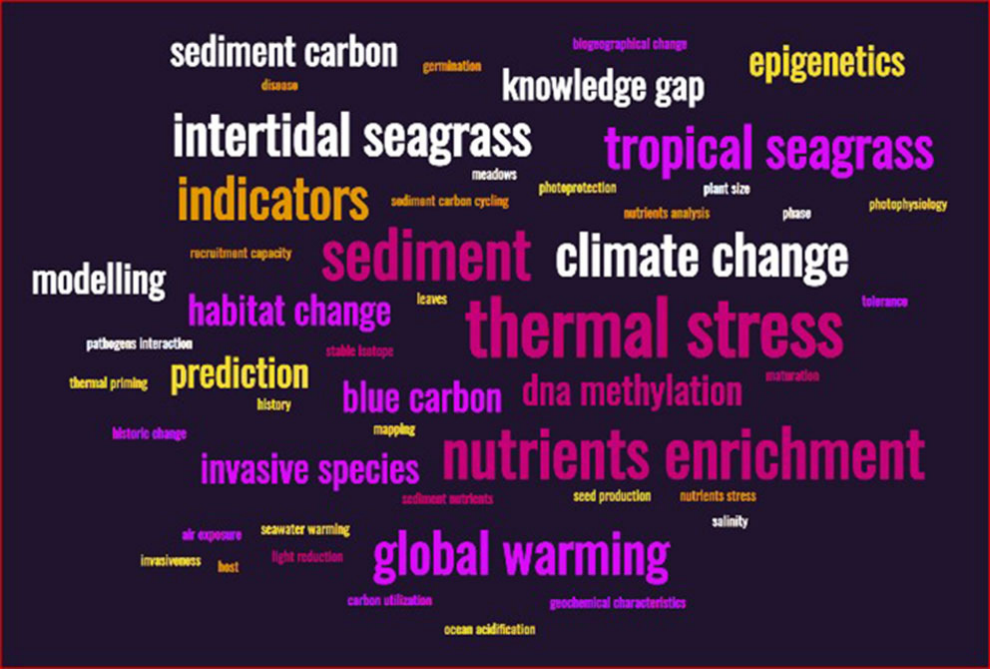
A word cloud representing the 4 prominent keywords used by each one of the 17 studies. The size of font/word is in relation to the number of times that term/word was repeated. We realize that some words are related/similar, but this is because of the word choice is made by a paper's authors, and thus thought all original words should be included as is.

In conclusion, extreme thermal events (e.g., heat waves or increasing seawater average temperature) and eutrophication-related stressors, such as light attenuation or nutrient increase, were, among others, the two main factors studied in this Research Topic. They both caused responses in photo-physiology, morphology, and tissue nutrient content in the diverse tropical and temperate species studied. Studies in this Research Topic have highlighted that coexisting species (even in the same meadow) assert different responses to stress or resilience capacity due to their different life-history traits. Moreover, it was shown that the same species growing in different environments or coming from different geographic areas can have contrasting responses to the same stress. Therefore, the conservation and management of seagrass meadows will depend on local studies that focus on the responses and resilience of the specific species and populations in the area. This Research Topic has also demonstrated high levels of plasticity exhibited by certain species to adverse environmental conditions, and that regular and consistent long-term monitoring of seagrass sites is needed to detect significant declines and plan conservation policies. Modeling species distribution under future temperature and salinity conditions project an increase in invasive species and a dramatic change of species composition in an exemplary study for the Mediterranean.

We hope that this Research Topic has not only answered some of the initial questions but has opened new research lines that generate a better understanding of seagrass loss in these changing times. This knowledge is needed to make effective decisions for the conservation of seagrass meadows worldwide.

## Author Contributions

GW initiated and led the writing. DW, MT, HR, and IV edited and improved earlier versions. All authors contributed to the article and approved the submitted version.

## Funding

This research was partially funded through the BMBF project SEANARIOS (Seagrass scenarios under thermal and nutrient stress: FKZ 03F0826A) to HR and MT. MT was partially funded through the DFG project SEAMAC (Seagrass and macroalgal community dynamics and performance under environmental change; TE 1046/3-1). IV was supported by a postdoctoral research grant Juan de la Cierva-Incorporación (IJC2019-040554-I) and from MCIN/AEI /10.13039/501100011033 (Spain).

## Conflict of Interest

The authors declare that the research was conducted in the absence of any commercial or financial relationships that could be construed as a potential conflict of interest.

## Publisher's Note

All claims expressed in this article are solely those of the authors and do not necessarily represent those of their affiliated organizations, or those of the publisher, the editors and the reviewers. Any product that may be evaluated in this article, or claim that may be made by its manufacturer, is not guaranteed or endorsed by the publisher.
